# The association between team resilience and team performance in nurses during COVID-19 pandemic: a network analysis

**DOI:** 10.1186/s12912-023-01216-w

**Published:** 2023-02-25

**Authors:** Zhiwei Wang, Qian Liang, Zeping Yan, Jian Liu, Mengqi Liu, Xiaoli Wang, Jiurui Wang, Junting Huang, Xiaorong Luan

**Affiliations:** 1 School of Nursing and Rehabilitation, Qilu Hospital, Shandong University, Shandong, China; 2 University of Health and Rehabilitation Sciences, Shandong, China; 3 School of Nursing, Midwifery and Health Systems, University College Dublin, Dublin, Ireland

**Keywords:** Team performance, Team resilience, Nurses, COVID-19, Network analysis

## Abstract

**Background:**

Compared to other healthcare workers, nurses are more vulnerable to the potentially devastating effects of pandemic-related stressors. Studies have not yet investigated the deeper characteristics of the relationship between team resilience and team performance among nurses during the COVID-19 pandemic. This study aimed to elucidate the characteristics of team resilience and performance networks among nurses during the pandemic.

**Methods:**

A cross-sectional study involving 118 nursing teams comprising 1627 practice nurses from four tertiary-A and secondary-A hospitals in Shandong Province, China, was conducted. Analyzing and Developing Adaptability and Performance in Teams to Enhance Resilience Scale and the Team Effectiveness Scale were used to measure team resilience and performance, respectively. The estimation of the network model and calculation of related metrics, network stability and accuracy, and network comparison tests were performed using R 4.0.2.

**Results:**

Node monitoring had the highest centralities in the team resilience and performance network model, followed by node anticipation, cooperation satisfaction, and cooperation with other departments. Moreover, node cooperation satisfaction and learning had the highest levels of bridge centrality in the entire network.

**Conclusion:**

Monitoring, anticipation, cooperation satisfaction, cooperation with other departments, and learning constituted core variables maintaining the team resilience-performance network structure of nurses during the pandemic. Clinical interventions targeting core variables may be effective in maintaining or promoting both team resilience and performance in this population.

**Supplementary Information:**

The online version contains supplementary material available at 10.1186/s12912-023-01216-w.

## Introduction

The coronavirus disease (COVID-19) pandemic has caused unprecedented public health challenges worldwide. There have been more than 161.5 million COVID-19 cases and more than 3.3 million deaths worldwide since the first cases were identified in China in December 2019 [1]. The high risk of infection and mortality because of the pandemic has caused a serious threat to the physical and mental health of healthcare workers, especially for nurses, who are closely involved in patient care [2, 3]. Nurses are front-line health workers and thus are constantly exposed to patients infected with the SARS-CoV-2 virus with little protection during the ongoing pandemic [2, 4]. Research has shown that nurses have had to face unique challenges because of the pandemic, including the threat of contracting COVID-19, unusually high workloads, and fear and worries related to being a carrier of the virus and infecting their family members [4]. Given these stressors, the psychological burden experienced by nurses is much higher than for other healthcare workers [5]. Team performance, which refers to the performance of the team at work [6], is affected by the mental wellbeing of team members. Specifically, the high psychological burden on nurses may lead to an impaired collective work [7], which might, in turn, jeopardize the safety of patients and the quality of care provided to them. For instance, a recent study noted that the working environment of healthcare teams is highly volatile and increasingly uncertain because of the nonstandard and complex working conditions resulting from the pandemic [8]. This significantly increases their chances of developing impaired work function (i.e., needle stick injuries, medication errors, and decreased work efficiency and patient satisfaction) [9]. Consequently, research focusing on the maintenance or improvement of team performance among nurses and their stress resistance during the pandemic is required [8, 10].

With the development of positive organizational behavior, researchers have begun to focus on the strengths and powers of teams, especially in the nursing scenario. Team resilience refers to a team’s ability to repair or bounce back from a potentially stressful situation [11]. It is an adaptation process in the face of adverse events that enables a team to better manage stress and thus maintain cohesion and performance [12]. Research has found that team resilience affects overall team performance and that those teams that have high levels of resilience are more likely to achieve positive team outcomes [13, 14]. However, it is worth noting that because of the complexity of the work environment, overall team performance is often evaluated by different structural components (e.g., task performance, cooperation satisfaction) [15], and there may be complex interactions among the structural components. Furthermore, different structural components of team resilience have various effects on team performance; for example, teams that have better knowledge about using resources have better overall performance than other structural components [16]. Therefore, previous studies on the relationship between team resilience and performance focusing only on the exploration of their overall relationship may not be comprehensive [13, 14]. To provide targeted coping strategies and/or interventions to improve team resilience and performance among nurses during the COVID-19 pandemic, it is important to understand the complex mechanisms between the components within and/or between team resilience and team performance.

In recent years, network analysis models have gained considerable attention as a new way of conceptualizing psychological phenomena in the field of psychological science. Rather than adding up the scores of potential variables (i.e., structural components) to describe psychological phenomena, network analysis is a potential variable-oriented approach that analyzes and documents the strength and nature of associations between potential variables from a mathematical point of view and efficiently visualizes them [17, 18]. This approach can provide the corresponding centrality and predictability indices for each potential variable and examine their importance and controllability in the overall network model [19, 20]. The central variable of the network may be regarded as a key target for the development of intervention strategies. Furthermore, network models can be used to identify bridge variables (i.e., variables that connect two psychological phenomena), which may play a role in the development and maintenance of symbiotic psychological phenomena [21]. Thus, researchers can tailor prevention or intervention strategies that simultaneously improve multiple psychological phenomena from the perspective of bridging variables. Network analysis models have been widely used in research in fields such as psychiatry and psychology [17, 18].

Recently, researchers have conducted network analyses of nurses’ work performance issues during the COVID-19 pandemic [22]. However, because nursing often requires effective teamwork [23], it is not comprehensive to analyze the network structure of nurses’ performance at an individual level, and thus, the network structure of nurse team performance should be analyzed separately. Furthermore, compared to other healthcare workers, nurses are more vulnerable to the potentially devastating effects of pandemic-related stressors [22]. Therefore, team resilience plays a pivotal role for them. If nurses have low levels of team resilience, it not only affects the overall performance of the team but is also detrimental to building a good nurse–patient relationship, reducing the effect of nursing intervention [24]. Thus, this study intends to i) examine the complex relationship between team resilience and team performance during the COVID-19 pandemic using network analysis among nurses; and ii) assess the potential impact of team grade on the observed network model; thereby providing a theoretical basis for developing targeted interventions for team resilience and team performance.

## Methods

### Study design and participants

The data for this cross-sectional study were collected via a web-based (WeChat-based “QuestionnaireStar” program) survey between September 2021 and October 2021 from practice nurses at four tertiary-A and secondary-A hospitals (based on hospital functions, scale, facilities, and technical level, the Health Administration of China classifies public hospitals into three levels, namely, primary hospitals, secondary hospitals, and tertiary hospitals, which are further divided into three subgroups, Grade A, Grade B, and Grade C. Tertiary-A hospitals represent the highest level of medical service in China), each in Shandong Province, China. On-site assessments were not adopted for convenience and safety reasons in light of the COVID-19 outbreak. Participants who met the following criteria were included in the study: (1) those who obtained the People’s Republic of China nursing qualification certificate and (2) those who worked in clinical nursing in a hospital. Nurses who were not involved in bedside care were also excluded. The beginning of the questionnaire included informed consent, and participants were informed of the purpose of the study before responding to the questionnaire. Only after providing informed consent could the participants access the questionnaire. In this study, the nurse team refers to the work team composed of multiple nurses in the same department or ward to jointly complete the task of patient care [15].

### Instrument

General data, including gender, age, education level, marital status, team size, and team grade (the ability of the nurse team to provide health care, which varies by level of hospital) were collected.

Team resilience was measured using the Analyzing and Developing Adaptability and Performance in Teams to Enhance Resilience Scale (ADAPTER) [25], which covers six essential capabilities of team resilience: responding, monitoring, learning, shared transformational leadership, cooperation with other departments, and anticipation. Each item on the ADAPTER was rated from 1 = “strongly disagree” to 5 = “strongly agree.” Higher total scores reflected greater team resilience. In this study, Cronbach’s alpha was calculated as 0.993 for the whole scale and between 0.919 and 0.986 for different dimensions.

Team performance was assessed using the Team Effectiveness Scale (TES) [26]. The TES comprises two dimensions: task performance and cooperation satisfaction. Each item on the TES is rated from 1 (“strongly disagree”) to 5 (“strongly agree”), and higher total scores indicate greater team performance. In this study, Cronbach’s alpha was calculated as 0.971 for the TES and 0.958 to 0.968 for the two dimensions.

### Data collection

Before the data collection process could advance, we contacted the nursing department of each sample hospital to request the electronic distribution of the questionnaire to nurses from all hospital departments or wards. After approving the request, the nursing department was formally asked to send online management questionnaires to the nurses via a WeChat group (Tencent Holdings Limited). During data collection, the authors regularly sent reminders to the nursing department when a decline in response rates was observed, in order to enhance response rates.

### Statistical analysis

#### Aggregation analysis

For the analysis, we first aggregated the individual scores at the team level. This is because team resilience and performance represent the shared perception of team members’ beliefs and attitudes. To justify data aggregation at the team level of analysis, the r_wg_ (acceptable range > 0.7) was calculated to assess the inter-rater agreement. The intraclass correlation coefficients (ICC) (1) (acceptable range > 0.05) and (ICC) (2) (acceptable range > 0.5) were calculated to assess the intraclass correlations and the reliability of the group means [27, 28].

#### Network analysis

Statistical analyses were performed using R 4.0.2. The network structure of team resilience and performance was established using the extended Bayesian information criterion (EBIC) graphical least absolute shrinkage and selection operator (LASSO) network models, and the network was estimated and visualized using the R packages qgraph and bootnet [29, 30]. The network layout has two main components: nodes (i.e., variables) and edges (i.e., each pairwise association between variables). Partial correlation analysis was used to calculate the association between each pairwise variable after controlling for other variables in the network [29]. A thicker edge represents a stronger association between two variables. Red and purple edges represented negative and positive associations, respectively.

To compare the importance of each variable within the model, three network centrality indices–betweenness, closeness, and strength–were investigated [31]. However, given that Bringmann et al. (2019) concluded that betweenness and closeness centrality are not appropriate indices for reflecting the importance of a variable in the psychological network, strength was the only centrality index to judge whether a variable was a core variable in this study [32]. Strength is the sum of the edge weights directly connected to a node. The R package MGM (mixed graphical model) was used to compute the predictability of each node. Predictability reflects the extent to which the variance of a node can be explained by other connected nodes [20]. Moreover, to identify bridge variables in the network, the bridge centrality index (i.e., bridge strength) was estimated using the R package network tools [21]. Bridge centrality reflects the possible connection modes between different communities (i.e., team resilience and performance) in the network. The higher the bridge centrality, the closer the connection between the bridge variable and all variables of other communities in the network [33].

#### Network stability and accuracy

The R package bootnet was used to examine the robustness of the network [29]. First, using a non-parametric bootstrap approach (1000 bootstrap samples), the accuracy of the edge weights was evaluated by calculating the 95% confidence intervals (CIs). Second, using a case-dropping bootstrap approach, the stability of node strength was assessed by computing the correlation-stability coefficient (CS-C). As indicated by Epskamp et al. (2018) [29], the value of CS-C should preferably be > 0.5 and should not be < 0.25. Third, bootstrapped difference tests (1000 bootstrap samples) of edge weights and node strengths were performed to examine differences in network properties.

#### Comparisons of network characteristics by team grade

To evaluate whether network characteristics differ as a function of team grade (tertiary versus secondary hospital nurse team), a network comparison test (NCT) was performed using the R package NetworkComparisonTest [34]. First, the distribution of the edge weights and the absolute sum of all edge weights in each network were compared to assess the network structure and global network strengths. Thereafter, the strength of each edge for networks between tertiary-A and secondary-A hospital nurse teams was examined using Holm–Bonferroni correlation for multiple comparisons.

### Ethical considerations

This study was performed in accordance with the Declaration of Helsinki and was approved by the Ethics Committee of Scientific Research of Shandong University Qilu Hospital (Ethics Approval No. KYLL-2020–258). Furthermore, all the information obtained from the participants remained strictly confidential and anonymous.

## Results

### Study sample

The study sample comprised a total of 118 nurse teams comprising 1627 nurses who completed the ADAPTER and TES. Of these, 56.8% and 43.2% were from tertiary-A and secondary-A hospitals, respectively. Their ages ranged from 19 to 57 years, with a mean age of 32.11 years and a standard deviation (SD) of ± 6.35. The number of members for each team was between 9 and 30. The team resilience and performance scores of the nurses in terms of different demographic characteristics are shown in Supplementary Table S1. The r_wg_, ICC (1), and ICC (2) values for team resilience and performance indicated that the aggregation of data to the team level was appropriate. The mean scores, abbreviations, r_wg_, ICC (1), and ICC (2) for each variable of ADAPTER and TES are shown in Table 1.

**Table 1:** Mean scores, abbreviations, r_wg_, ICC (1), ICC (2), and predictability for each variable of ADAPTER and TES (*N* = 118 teams)

Variables	Abbreviation	r_wg_	ICC (1)	ICC (2)	Mean (SD)	Predictability
Team resilience (ADAPTER)						
Responding	Responding	0.97	0.05	0.55	36.00 (1.90)	70.7%
Shared transformational leadership	Leadership	0.98	0.07	0.62	58.69 (3.20)	94.7%
Learning	Learning	0.92	0.05	0.57	17.68 (1.05)	85.8%
Anticipating	Anticipating	0.96	0.07	0.62	22.69 (1.18)	97.0%
Monitoring	Monitoring	0.98	0.06	0.60	45.35 (2.32)	97.7%
Cooperation with other departments	Cooperation	0.98	0.06	0.61	36.28 (1.87)	96.3%
Team performance (TES)						
Task performance	Task performance	0.95	0.06	0.59	22.27 (1.26)	88.5%
Cooperation satisfaction	Cooperation satisfaction	0.93	0.06	0.58	13.63 (0.72)	90.6%

*ADAPTER* Analysing and Developing Adaptability and Performance in Teams to Enhance Resilience Scale, *TES* Team Effectiveness Scale, *r*_*wg*_ interrater agreement index, *ICC* intraclass correlation coefficient, *SD* standard deviation

### Network structure

**Fig. 1:**
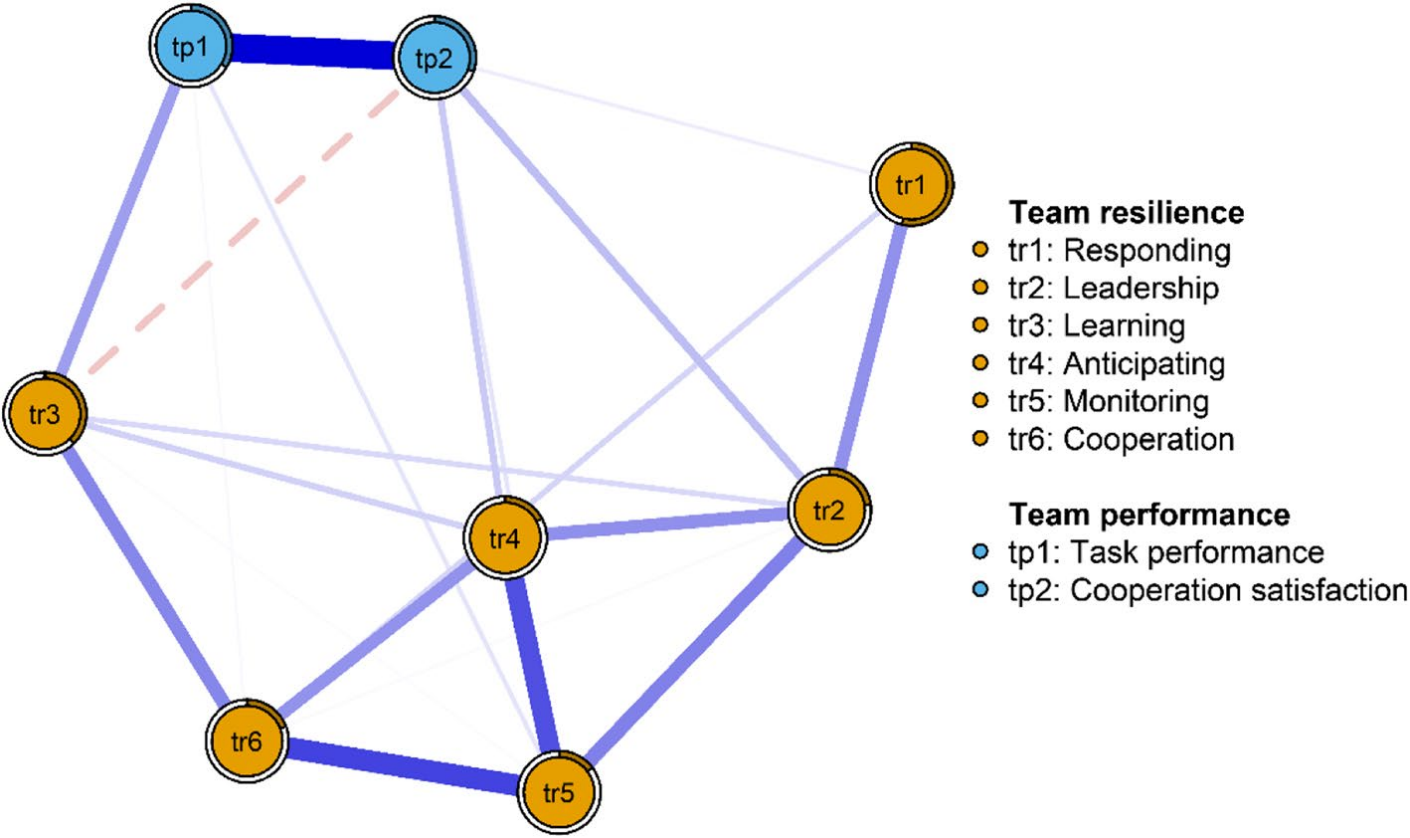
Network structure of team resilience and performance among nurses

A network of team resilience and performance is shown in Fig. 1. A total of 21 edges from 28 were above zero, and all these connections were positive associations, barring the learning cooperation satisfaction edge. In the team resilience network model, the monitoring-cooperation edge had the strongest connection, followed by the anticipating, leadership, and learning cooperation edges. Within the team performance network model, task performance had a strong connection with cooperation satisfaction. In the team resilience and performance network model, the learning-task performance edge showed the strongest connection between team resilience and performance. The correlation matrices for each node of team resilience and team performance network are shown in Supplementary Table S2.

**Fig. 2:**
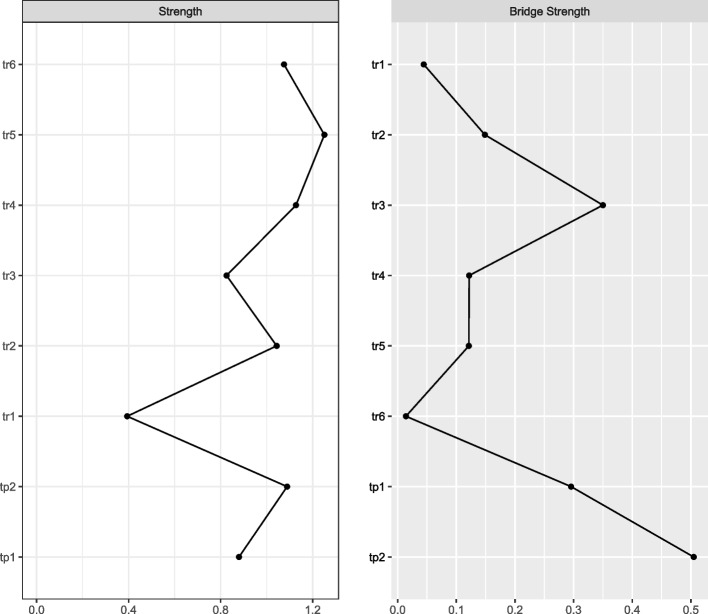
Centrality indices (left) and bridge centrality indices (right) of network structure of team resilience and performance among nurses

In terms of network centrality, node monitoring had the highest strength, followed by node anticipation, cooperation satisfaction, and cooperation (see Fig. 2), all of which emerged as core variables in understanding the team resilience and team performance network in nurses. Contrastingly, the node response had the lowest strength. According to the bridge strength shown in Fig. 2, the results identified cooperation satisfaction and learning as the two most prominent bridge variables.

Regarding the predictability of each node, the average was 90.2%, indicating that 90.2% of the variance of each variable was explained by other connected variables. Monitoring (97.7%) in the network had the highest predictability, followed by anticipation (97.0%), and cooperation (96.3%) (see Table 1). The predictability of each node is shown as a circle around it in Fig. 1.

### Network stability and accuracy

**Fig. 3:**
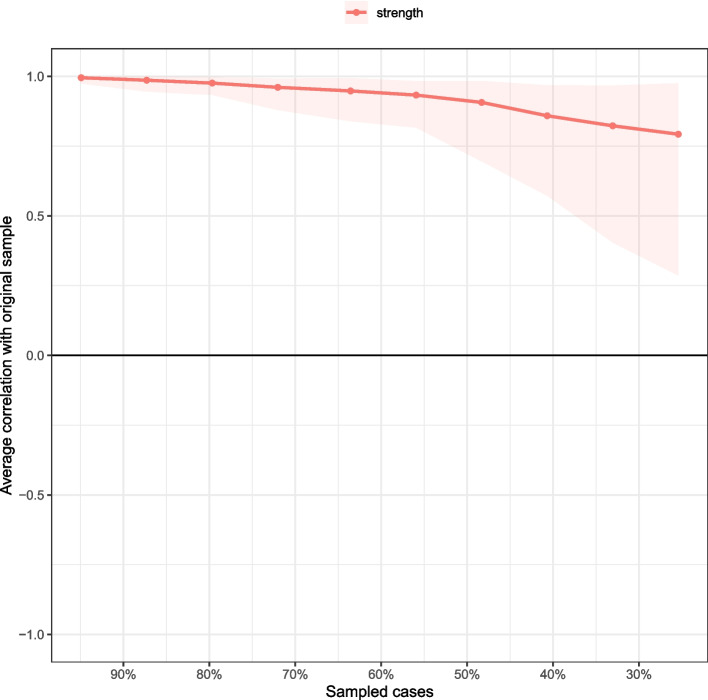
The stability of network structure by case dropping subset bootstrap

The case-dropping bootstrap procedure for the node strength is shown in Fig. 3. The CS-C for node strength was 0.517, indicating that the network model was adequately stable (i.e., 51.7% of the sample could be dropped without significant changes compared to the primary results). The results of bootstrapped 95% CIs for edge weights implied that the network model was reliable (Supplementary Figure S1). The results of the bootstrapped difference tests for edge weights and node strength are presented in the supplementary materials (Figures S2-S3).

### Comparisons based on team grade

In the NCT of networks between tertiary-A and secondary-A hospital nurse teams, no significant differences were found in network structure (M = 0.24, *p* = 0.755), network global strength (tertiary-A hospital nurse teams: 4.02 vs secondary-A hospital nurse teams: 3.78; S = 0.24, *p* = 0.606), or strength for each edge of network (all *p* > 0.05). The plots are shown in Supplementary Figure S4.

## Discussion

To the best of our knowledge, this is the first study to characterize the team resilience and performance network of nurses during the COVID-19 crisis. The present analyses showed that node monitoring had the highest centralities in the entire network, followed by node anticipation, cooperation satisfaction, and cooperation, all of which emerged as core variables in understanding the team resilience and team performance network of nurses. Node cooperation satisfaction and learning had the highest levels of bridge centrality in the entire network, which were considered as bridge variables linking team resilience and performance. Additionally, some important strong edges identified in the current network were the connections between node monitoring and cooperation, node anticipation and monitoring, and leadership and monitoring.

Network analysis revealed that monitoring was the most urgently needed expertise of nurse teams during the COVID-19 crisis. Monitoring is defined as the capability of team members to monitor each other’s or the team’s performance, as well as what happens when faced with unexpected situations [25, 35], which helps promote team effectiveness in unexpected situations. The pandemic is an unexpected situation for nursing teams. Therefore, to maintain or promote team performance, monitoring is unequivocally necessary for nurse teams during the COVID-19 pandemic. On the one hand, nurses can monitor the performance of a team to which they belong to gain insight into their team’s mishandling of work processes and correct the resulting errors; on the other hand, nurses can circumvent underperformance by self-reflecting on their own performance (i.e., self-monitoring), such as “what went well” and “what could have done better” [36]. Furthermore, previous research has found that team performance is positively associated with teamwork quality, and monitoring is an important prerequisite for effective teamwork [37, 38]. Thus, to improve team performance among nurses during the COVID-19 pandemic, monitoring may indeed be the most urgently needed ability for nurse teams, and this may be a critical intervention target for nurse managers to improve both team resilience and team performance among nurses during the COVID-19 pandemic.

Anticipating was identified as another urgently needed capability for nurse teams in this study. As the pandemic led to nonstandard and complex working conditions, nurses have experienced increased uncertainty in the course of their work [8]. To ensure quality of care and patient safety, it is necessary to develop nurses’ anticipating capacity so that they are more adept at making accurate judgments about unexpected situations. Furthermore, cooperation satisfaction and cooperation were found to be the core variables in the team resilience and performance network of nurses. This finding was similar to that of Schmutz et al. (2019) [39], who concluded that cooperation is a critical capability for healthcare teams to maintain or promote team performance in unexpected situations. As such, strengthening nurses’ cooperation capability should be incorporated within programs designed to improve team resilience and performance.

There are two strong bridge variables in this study: cooperation satisfaction and learning. These findings suggest that when team performance is poor, interventions targeting “cooperation satisfaction” will not only improve team performance but may also improve overall team resilience. However, when team resilience is poor, interventions targeting “learning” will not only improve team resilience but may, in turn, improve overall team performance. Previous studies on the influences of team performance and team resilience found that cooperation satisfaction was a critical factor affecting team performance and team resilience, which could substantiate our findings [14, 40]. Moreover, in studies assessing team performance and resilience [16, 25, 26], cooperation is an evaluation criterion for team performance and resilience in company workers. As for “learning,” it is a critical factor that affects not only team resilience but also team performance among healthcare staff [41, 42]. Given the significant differences in team characteristics and work contexts among the participants, the above variables are plausible candidates as signature bridge variables for simultaneous improvements in team resilience and performance among nurses.

The current study found some strong connections between “monitoring” and “cooperation,” between “anticipating” and “monitoring,” and between “leadership” and “monitoring;” all these connections underscore the important relationship between monitoring and other team capabilities. To the best of our knowledge, this finding has not been previously reported and may be unique to our study sample (i.e., Chinese nursing teams); this requires further investigation. Indeed, there is a strict hierarchy of leadership between superiors and subordinates because of the traditional nursing management culture in China (hospitals in China have a hierarchical management system for nurses, that is, based on clinical nursing competency, hospitals divide nurses into different hierarchical levels. High-hierarchical-level nurses often play leading roles in work, whereas low-hierarchical-level nurses carry out high-hierarchical-level nurses’ orders) [43]. For nurse teams in each department, the process of cooperation within the team, the process of honing the anticipating capability of the team, and the leadership style within the team are all monitored by the supervisors. Moreover, the study was conducted in Shandong Province, China, which has deeper roots in hierarchical management culture [43]. That is, staff from Shandong were more powerful than those from other regions, and in this case, the process of cooperation within the team, the process of honing the anticipating capability of the team, and the leadership style within the team tends to be more strictly monitored by supervisors. Furthermore, these findings may provide several potential intervention pathways to improve the monitoring capability of nurse teams. The predictability of “monitoring” was found to be 97.7%, suggesting that it is highly influenced by its neighboring variables in the current network. This finding suggests that intervention in “monitoring” can be achieved not only by intervening in “monitoring” itself or by affecting other relevant factors not included in the current network but also by intervening on strong neighboring variables (i.e., “cooperation,” “anticipating,” and “leadership”).

This study has several strengths. First, this was a multicenter study and the results might be more generalizable than those of a single-center study. Second, the team resilience and performance network had adequate stability and accuracy, as shown by bootstrapping analyses. Furthermore, the NCT analysis showed that team resilience and team performance network structure did not vary by team grade.

In addition to the strengths mentioned above, this study also has several limitations. First, because this study used cross-sectional data to build an observed network model, it was not possible to infer a causal relationship between the nodes of team resilience and team performance. Longitudinal data can be used to assess the dynamics between nodes of team resilience and team performance over time. Second, the current findings are limited to Chinese nursing teams in a pandemic scenario. Hence, team resilience and performance networks in non-pandemic contexts and/or other populations may differ from the network structure of this study. Finally, the variable patterns of team resilience and performance in this study were specific to the instruments used for analysis. There is often a discrepancy among self-report tools for assessing variables of team resilience and team performance. Therefore, different assessment tools can lead to different network structures.

## Conclusions

To conclude, this study revealed that monitoring, anticipation, cooperation satisfaction, and cooperation are the most urgently needed capabilities for nursing teams and can be regarded as potential clinical intervention targets for maintaining or promoting both team resilience and team performance among nurses during the COVID-19 pandemic. Interventions targeting bridge variables (cooperation satisfaction and learning) may contribute to simultaneously improving team resilience and performance. These results also identify a strong connection between monitoring and most other team capabilities (cooperation, anticipation, and leadership), which requires further research.

## Implications for nursing management

Recently, the issue of team performance during a pandemic has been the focus of nursing management. To solve this issue, understanding the dynamic and reciprocal relationships between team resilience and performance, especially during pandemics, is undoubtedly important. Nurse managers and hospital administrators can use the network to gain insight into the relationships within and between communities (i.e., team resilience and team performance), thereby tailoring interventions that maintain or improve nurses’ collective stress resistance and performance and improving the quality of nursing services.

Specifically, nurse managers and hospital administrators should consider investment in improving the capability of nurses to monitor each other’s or the team’s performance during the COVID-19 crisis: for example, organizing regular seminars or team meetings, characterized by active listening and effective communication, and facilitating nurses’ communication and reflection on “what went well” and “what could have done better” to look for positive work practices [44]. In addition, developing the capability of nurses to anticipate unexpected situations during the COVID-19 crisis should also be considered. On the one hand, managers can organize regular in-situ simulation trainings and debriefings, characterized by managing different situations and cases, helping nurses understand practice and adapt to routine and unexpected situations, thereby building the best coping strategies that foster the development of resilient behaviors, such as anticipating, when facing similar situations. On the other hand, managers can mix experienced and inexperienced people to enable experienced nurses to teach novices how the health care system works and how the organization adapts to and copes with expected and unexpected situations, allowing them to take resilient action proactively in routine and unexpected situations [44]. Additionally, establishing an environment that encourages cooperation and learning among nurses can benefit nurses greatly. Therefore, in the course of their daily work, managers can encourage nurses to participate in and contribute to cooperation and learning by making it an integral part of their evaluations in order to create a positive cooperation and learning climate.

Hospital education departments can also use this information to design curricula for nurses to manage stress and performance during health crises and provide effective care to patients who are also affected by these crises. In addition, social or health policymakers can use this information to develop support programs or improve existing programs focused on stress management for nurses.

## Supplementary Information

**Additional file 1:** **Supplementary Table 1.** Scores of team resilience and team performance in relation to demographic characteristics. **Supplementary Table 2.** Correlation matrices for each node of team resilience and team performance network. **Supplementary Figure 1.** Bootstrapped 95% confidence intervals (CIs) of edge weights. **Supplementary Figure 2.** Bootstrapped difference test for edge weights. **Supplementary Figure 3.** Bootstrapped difference test for node strength. **Supplementary Figure 4.** Estimated network of team resilience and performance in tertiary-A (*N*=67)and secondary-A hospital nurse teams (*N*=51). 

## Data Availability

The datasets generated and/or analyzed during the current study are not publicly available due to privacy or ethical restrictions but are available from the corresponding author on reasonable request.

## Abbreviations

ADAPTER: Analyzing and developing adaptability and performance in teams to enhance resilience scale
TES: Team effectiveness scale
r_wg_: Interrater agreement index
ICC: Intraclass correlation coefficient
CIs: Confidence intervals
CS-C: Correlation-stability coefficient
NCT: Network comparison test
SD: Standard deviation

## References

[1] WHO. Coronavirus (COVID-19) Dashboard. Available from: https://covid19.who.int/.

[2] Baysal E, Selcuk AK, Aktan GG, Andrade EF, Notarnicola I, Stievano A (2022). An examination of the fear of COVID-19 and professional quality of life among nurses: a multicultural study. J Nurs Manage.

[3] Serrano-Ripoll MJ, Meneses-Echavez JF, Ricci-Cabello I, Fraile-Navarro D, Fiol-deRoque MA, Pastor-Moreno G (2020). Impact of viral epidemic outbreaks on mental health of healthcare workers: A rapid systematic review and meta-analysis. J Affect Disorders.

[4] Spoorthy MS, Pratapa SK, Mahant S (2020). Mental health problems faced by healthcare workers due to the COVID-19 pandemic-a review. Asian J Psychiatr.

[5] Kang LJ, Li Y, Hu SH, Chen M, Yang C, Yang BX (2020). The mental health of medical workers in Wuhan, China dealing with the 2019 novel coronavirus. Lancet Psychiat.

[6] Gladstein DL (1984). Groups in context: A model of task group effectiveness. Admin Sci Quart.

[7] Dietz AS, Driskell JE, Sierra MJ, Weaver SJ, Driskell T, Salas E, Salas E, Rico R, Passmore J (2017). Teamwork under stress. The Wiley Blackwell handbook of the psychology of team working and collaborative processes.

[8] Joniakova Z, Jankelova N, Blstakova J, Nemethova I (2021). Cognitive diversity as the quality of leadership in crisis: Team performance in health service during the COVID-19 pandemic. Healthcare.

[9] Gartner FR, Nieuwenhuijsen K, van Dijk FJH, Sluiter JK (2010). The impact of common mental disorders on the work functioning of nurses and allied health professionals: a systematic review. Int J Nurs Stud.

[10] Lieslehto J, Rantanen N, Oksanen LMAH, Oksanen SA, Kivimaki A, Paju S (2022). A machine learning approach to predict resilience and sickness absence in the healthcare workforce during the COVID-19 pandemic. Sci Rep.

[11] West BJ, Patera JL, Carsten MK (2009). Team level positivity: Investigating positive psychological capacities and team level outcomes. J Organ Behav.

[12] Hartwig A, Clarke S, Johnson S, Willis S (2020). Workplace team resilience: a systematic review and conceptual development. Organ Psychol Rev.

[13] Dimas ID, Rebelo T, Lourenco PR, Pessoa CIP (2018). Bouncing back from setbacks: On the mediating role of team resilience in the relationship between transformational leadership and team effectiveness. J Psychol.

[14] Meneghel I, Salanova M, Martinez IM (2016). Feeling good makes us stronger: How team resilience mediates the effect of positive emotions on team performance. J Happiness Stud.

[15] Feng T, Su JK (2021). Study on the relationship between nursing transformational leadership and team performance in military hospital. Mil Med J S Chin.

[16] McEwen K, Boyd CM (2018). A measure of team resilience: developing the resilience at work team scale. J Occup Environ Med.

[17] Beard C, Millner AJ, Forgeard MJC, Fried EI, Hsu KJ, Treadway MT (2016). Network analysis of depression and anxiety symptom relationships in a psychiatric sample. Psychol Med.

[18] Rouquette A, Pingault JB, Fried EI, Orri M, Falissard B, Kossakowski JJ (2018). Emotional and behavioral symptom network structure in elementary school girls and association with anxiety disorders and depression in adolescence and early adulthood: a network analysis. Jama Psychiat.

[19] Contreras A, Nieto I, Valiente C, Espinosa R, Vazquez C (2019). The study of psychopathology from the network analysis perspective: a systematic review. Psychother Psychosom.

[20] Haslbeck JMB, Fried EI (2017). How predictable are symptoms in psychopathological networks? A reanalysis of 18 published datasets. Psychol Med.

[21] Jones PJ, Ma RF, McNally RJ (2021). Bridge centrality: a network approach to understanding comorbidity. Multivar Behav Res.

[22] Tokac U, Razon S (2021). Nursing professionals’ mental well-being and workplace impairment during the COVID-19 crisis: a network analysis. J Nurs Manage.

[23] Meyers DJ, Chien AT, Nguyen KH, Li ZH, Singer SJ, Rosenthal MB (2019). Association of team-based primary care with health care utilization and costs among chronically III patients. Jama Intern Med.

[24] Son DM, Ham OK (2020). Influence of group resilience on job satisfaction among Korean nurses: a cross-sectional study. J Clin Nurs.

[25] van der Beek D, Schraagen JM (2015). ADAPTER: Analysing and developing adaptability and performance in teams to enhance resilience. Reliab Eng Syst Safe.

[26] Tjosvold D (1988). Cooperative and competitive interdependence: collaboration between departments to serve customers. Group Organ Stud.

[27] Bliese PD, Klein KJ, Kozlowski SWJ (2000). Within-group agreement, non-independence, and reliability: Implications for data aggregation and analysis. Multilevel theory, research, and methods in organizations.

[28] Glick WH (1985). Conceptualizing and measuring organizational and psychological climate: Pitfalls in multilevel research. Acad Manage Rev.

[29] Epskamp S, Borsboom D, Fried EI (2018). Estimating psychological networks and their accuracy: a tutorial paper. Behav Res Methods.

[30] Epskamp S, Cramer AOJ, Waldorp LJ, Schmittmann VD, Borsboom D (2012). Qgraph: network visualizations of relationships in psychometric data. J Stat Softw.

[31] Opsahl T, Agneessens F, Skvoretz J (2010). Node centrality in weighted networks: generalizing degree and shortest paths. Soc Networks.

[32] Bringmann LF, Elmer T, Epskamp S, Krause RW, Schoch D, Wichers M (2019). What do centrality measures measure in psychological networks?. J Abnorm Psychol.

[33] Wang YY, Hu ZS, Feng Y, Wilson A, Chen RS (2020). Changes in network centrality of psychopathology symptoms between the COVID-19 outbreak and after peak. Mol Psychiatr.

[34] van Borkulo CD, Epskamp S, Millner A. Network Comparison Test: Permutation-based test of differences in strength of networks. Available from: https://cran.r-project.org/web/packages/NetworkComparisonTest/NetworkComparisonTest.pdf.

[35] Hollnagel E. Epilogue: RAG—the resilience analysis grid. In: Hollnagel E, Paries J, Woods D, Wreathall J, editors. Resilience engineering in practice: a guidebook. Aldershot: Ashgate; 2011. p. 275–96.

[36] Hautz SC, Oberholzer DL, Freytag J, Exadaktylos A, Kammer JE, Sauter TC (2020). An observational study of self-monitoring in ad hoc health care teams. Bmc Med Educ.

[37] Manser T (2009). Teamwork and patient safety in dynamic domains of healthcare: a review of the literature. Acta Anaesth Scand.

[38] Salas E, Sims DE, Burke CS (2005). Is there a “big five” in teamwork?. Small Gr Res.

[39] Schmutz JB, Meier LL, Manser T (2019). How effective is teamwork really? The relationship between teamwork and performance in healthcare teams: a systematic review and meta-analysis. BMJ Open.

[40] Song H, Ryan M, Tendulkar S, Fisher J, Martin J, Peters AS (2017). Team dynamics, clinical work satisfaction, and patient care coordination between primary care providers: a mixed methods study. Health Care Manage R.

[41] Dubois CA, Da Silva RB, Lavoie-Tremblay M, Lesperance B, Bentein K, Marchand A (2020). Developing and maintaining the resilience of interdisciplinary cancer care teams: an interventional study. Bmc Health Serv Res.

[42] Rosen MA, DiazGranados D, Dietz AS, Benishek LE, Thompson D, Pronovost PJ (2018). Teamwork in healthcare: Key discoveries enabling safer, high-quality care. Am Psychol.

[43] Yu HP, Zhang WY, Peng YQ, Hung YY, Chen C, Li YY (2020). Emergency medical staff’s perceptions on cultural value difference-based teamwork issues: a phenomenological study in China. J Nurs Manage.

[44] Iflaifel M, Lim RH, Ryan K, Crowley C (2020). Resilient health care: a systematic review of conceptualisations, study methods and factors that develop resilience. Bmc Health Serv Res.

