# Supplementary material for: The association between team resilience and team performance in nurses during COVID-19 pandemic: a network analysis
Source: BMC Nurs. 2023 Feb 25;22:54. doi: 10.1186/s12912-023-01216-w (PMC9959955; doi:10.1186/s12912-023-01216-w)
Supplement: Supplementary file 1 — Additional file 1: Supplementary Table 1. Scores of team resilience and team performance in relation to demographic characteristics. Supplementary Table 2. Correlation matrices for each node of team resilience and team performance network. Supplementary Figure 1. Bootstrapped 95% confidence intervals (CIs) of edge weights. Supplementary Figure 2. Bootstrapped difference test for edge weights. Supplementary Figure 3. Bootstrapped difference test for node strength. Supplementary Figure 4. Estimated network of team resilience and performance in tertiary-A (N=67)and secondary-A hospital nurse teams (N=51). [file 12912_2023_1216_MOESM1_ESM.docx]

**Supplementary Table 1** Scores of team resilience and team performance in relation to demographic characteristics

| **Variable** | **Values** | **N** | **Team resilience** | |  | **Team performance** | |
| --- | --- | --- | --- | --- | --- | --- | --- |
|  |  |  | **Mean (SD)** | **P** |  | **Mean (SD)** | **P** |
| **Gender** | Female | 1523 | 216.78 (22.88) | 0.089* |  | 35.89 (4.82) | 0.424* |
|  | Male | 104 | 212.84 (23.27) |  |  | 35.50 (4.58) |  |
| **Education level** | Junior college degree | 225 | 223.55 (20.74) | < 0.001** |  | 36.91 (4.78) | 0.002** |
|  | Undergraduate degree | 1366 | 215.49 (23.19) |  |  | 35.70 (4.81) |  |
|  | Postgraduate degree | 36 | 212.17 (17.08) |  |  | 35.53 (3.92) |  |
| **Marital status** | Unmarried | 412 | 218.36 (22.29) | 0.171** |  | 36.05 (4.72) | 0.667** |
|  | Married | 1191 | 215.91 (23.16) |  |  | 35.80 (4.85) |  |
|  | Divorced/widowed | 24 | 215.88 (20.26) |  |  | 35.75 (3.92) |  |
| **Team grade** | Tertiary hospital | 67 | 213.14 (9.94) | < 0.001* |  | 35.26 (1.77) | < 0.001* |
|  | Secondary hospital | 51 | 221.33 (10.80) |  |  | 36.74 (1.87) |  |

*. T - test

**. ANOVA test

**Supplementary Table 2** Correlation matrices for each node of team resilience and team performance network

|  | Responding | Leadership | Learning | Anticipating | Monitoring | Cooperation | Task performance | Cooperation satisfaction |
| --- | --- | --- | --- | --- | --- | --- | --- | --- |
| Responding | 0.00 |  |  |  |  |  |  |  |
| Leadership | 0.25 | 0.00 |  |  |  |  |  |  |
| Learning | 0.00 | 0.09 | 0.00 |  |  |  |  |  |
| Anticipating | 0.00 | 0.25 | 0.10 | 0.00 |  |  |  |  |
| Monitoring | 0.00 | 0.29 | 0.01 | 0.40 | 0.00 |  |  |  |
| Cooperation | 0.10 | 0.01 | 0.28 | 0.25 | 0.43 | 0.00 |  |  |
| Task performance | 0.00 | 0.00 | 0.22 | 0.00 | 0.06 | 0.01 | 0.00 |  |
| Cooperation satisfaction | 0.04 | 0.15 | -0.13 | 0.12 | 0.06 | 0.00 | 0.58 | 0.00 |


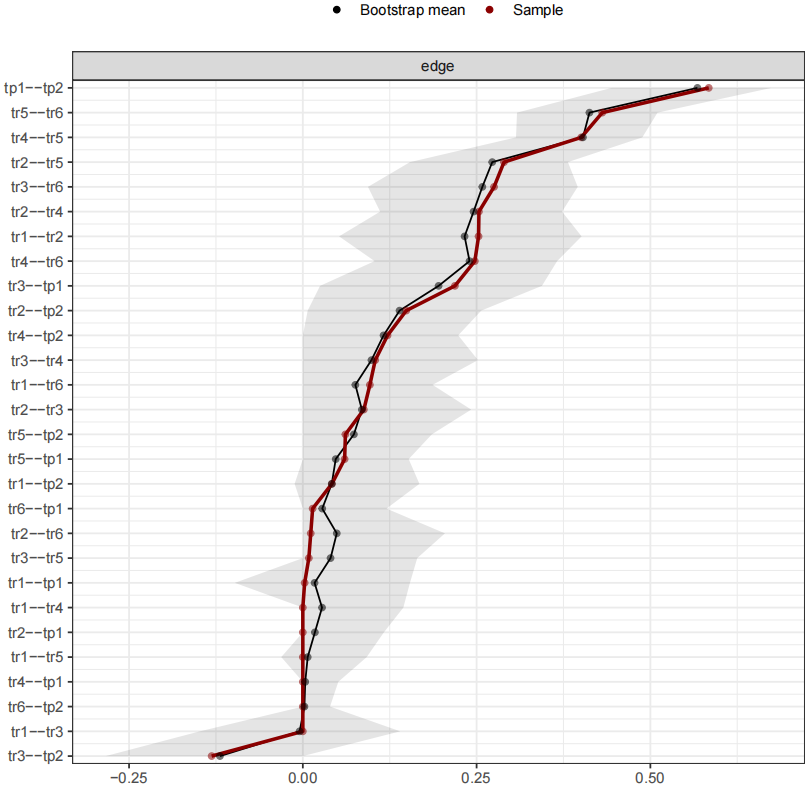


**Supplementary Figure 1** Bootstrapped 95% confidence intervals (CIs) of edge weights

*Note*: The red line represents the edge weights. The gray area depicts the 95%CIs of edge weights. (tr1: Responding, tr2: Leadership, tr3: Learning, tr4: Anticipating, tr5: Monitoring, tr6: Cooperation; tp1: Task performance, tp2: Cooperation satisfaction)


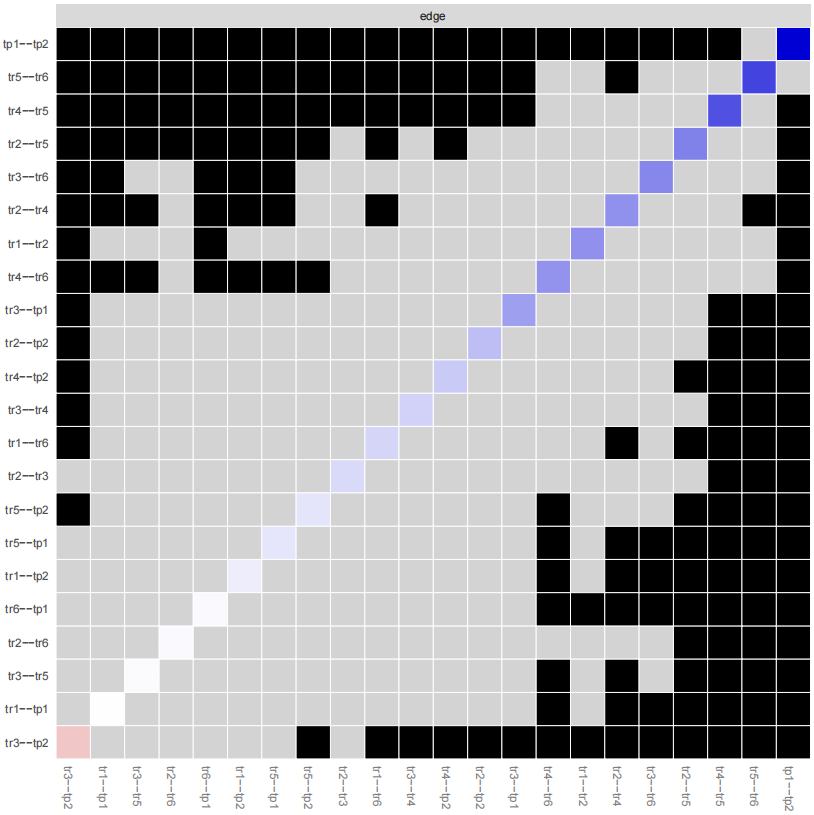


**Supplementary Figure 2** Bootstrapped difference test for edge weights

*Note*: Gray boxes represent no significant difference between edge weights, while black boxes represent significant difference. Blue boxes on the diagonal represent edge weights with positive correlations. Red boxes on the diagonal represent edge weights with negative correlations. (tr1: Responding, tr2: Leadership, tr3: Learning, tr4: Anticipating, tr5: Monitoring, tr6: Cooperation; tp1: Task performance, tp2: Cooperation satisfaction)


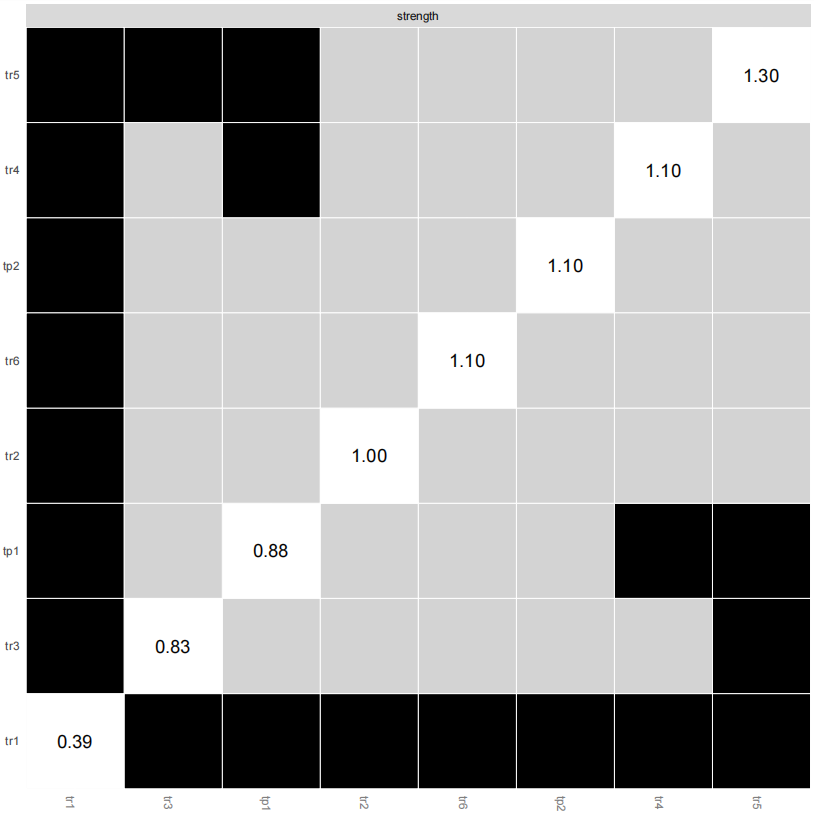


**Supplementary Figure 3** Bootstrapped difference test for node strength

*Note*: Gray boxes represent no significant difference between nodes, while black boxes represent significant difference. White boxes on the diagonal represent the values of node strength. (tr1: Responding, tr2: Leadership, tr3: Learning, tr4: Anticipating, tr5: Monitoring, tr6: Cooperation; tp1: Task performance, tp2: Cooperation satisfaction)


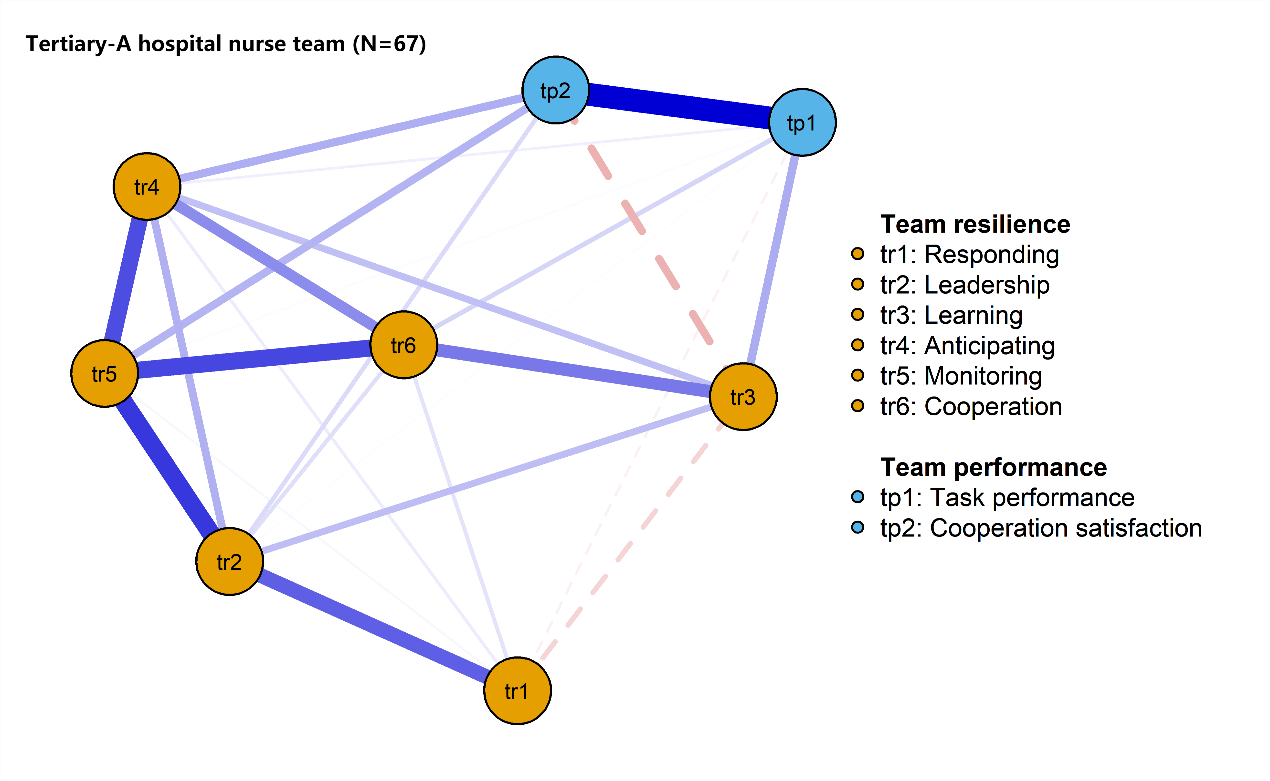


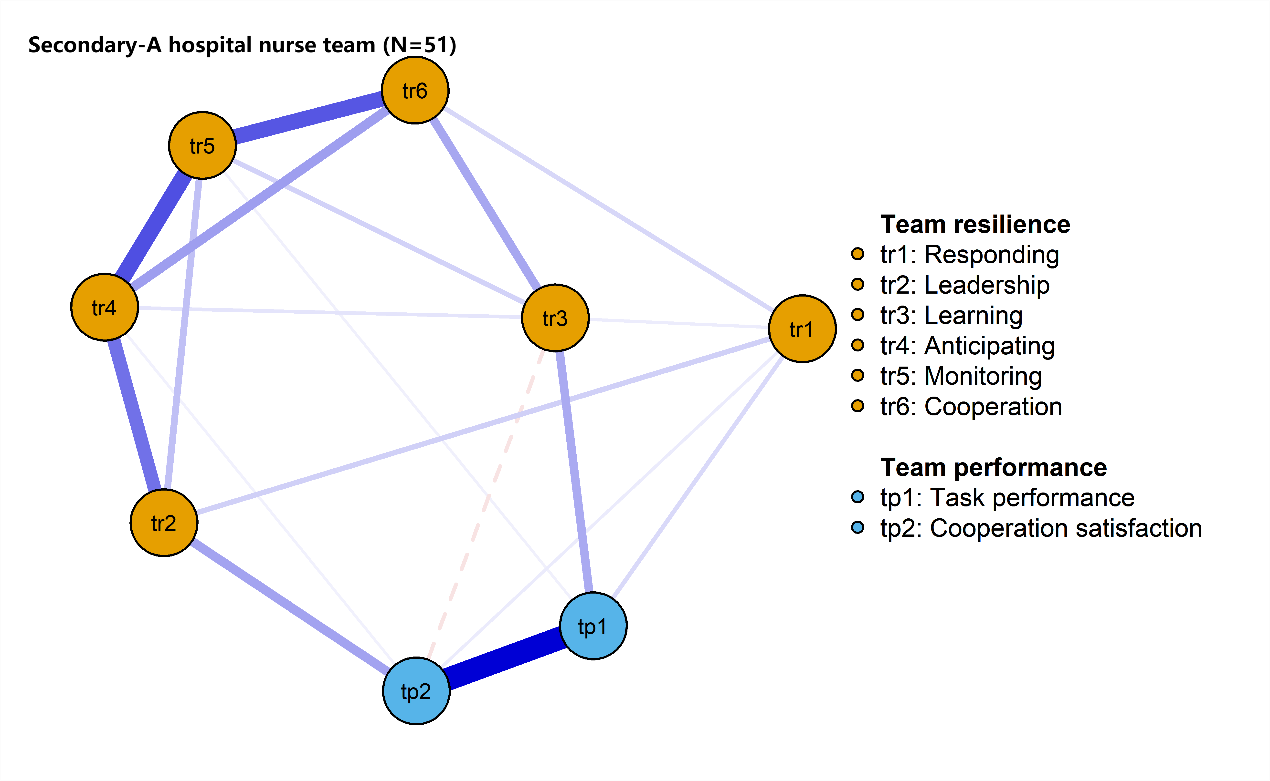


**Supplementary Figure 4** Estimated network of team resilience and performance in tertiary-A (N=67) and secondary-A hospital nurse teams (N=51)
